# MTA index: a simple 2D-method for assessing atrophy of the medial temporal lobe using clinically available neuroimaging

**DOI:** 10.3389/fnagi.2014.00023

**Published:** 2014-03-24

**Authors:** Manuel Menéndez-González, Alfonso López-Muñiz, José A. Vega, José M. Salas-Pacheco, Oscar Arias-Carrión

**Affiliations:** ^1^Unidad de Neurología, Hospital Álvarez-BuyllaMieres, Spain; ^2^Departamento de Morfología y Biología Celular, Universidad de OviedoOviedo, Spain; ^3^Instituto de Neurociencias, Universidad de OviedoOviedo, Spain; ^4^Instituto de Investigación Científica, Universidad Juárez del Estado de DurangoDurango, México; ^5^Unidad de Trastornos del Movimiento y Sueño (TMS), Hospital General Dr. Manuel Gea González/UNAMMéxico DF, Mexico; ^6^Unidad de Trastornos del Movimiento y Sueño (TMS), Hospital General Ajusco MedioMéxico DF, Mexico

**Keywords:** medial temporal lobe atrophy, biomarker, Alzheimer, mild cognitive impairment, MRI, neuroimaging, diagnosis

## Abstract

**Background and purpose:** Despite a strong correlation to severity of AD pathology, the measurement of medial temporal lobe atrophy (MTA) is not being widely used in daily clinical practice as a criterion in the diagnosis of prodromal and probable AD. This is mainly because the methods available to date are sophisticated and difficult to implement for routine use in most hospitals—volumetric methods—or lack objectivity—visual rating scales. In this pilot study we aim to describe a new, simple and objective method for measuring the rate of MTA in relation to the global atrophy using clinically available neuroimaging and describe the rationale behind this method.

**Description:** This method consists of calculating a ratio with the area of 3 regions traced manually on one single coronal MRI slide at the level of the interpeduncular fossa: (1) the medial temporal lobe (MTL) region (A); (2) the parenchima within the medial temporal region, that includes the hippocampus and the parahippocampal gyrus—the fimbria taenia and plexus choroideus are excluded—(B); and (3) the body of the ipsilateral lateral ventricle (C). Therefrom we can compute the ratio “Medial Temporal Atrophy index” at both sides as follows: *MTAi* = (*A* − *B*)× 10/*C*.

**Conclusions:** The MTAi is a simple 2D-method for measuring the relative extent of atrophy in the MTL in relation to the global brain atrophy. This method can be useful for a more accurate diagnosis of AD in routine clinical practice. Further studies are needed to assess the usefulness of MTAi in the diagnosis of early AD, in tracking the progression of AD and in the differential diagnosis of AD with other dementias.

## Background

Alzheimer's disease's (AD) pathology accumulates for years and may be even decades before it is typically diagnosed (Morris et al., 1996). Sensitive biomarker techniques may be able to pick up signs of neurodegeneration presymptomatically. Recently proposed criteria for research purposes for prodromal AD (Sperling et al., 2011), mild cognitive impairment (MCI) due to AD (Albert et al., 2011), and probable AD dementia (McKhann et al., 2011) incorporate evidence of AD pathology including molecular changes and brain structure and function as supportive biomarkers. MRI-based biomarkers are among the supportive evidence for a diagnosis of early AD and MCI due to AD. By focusing on cortical regions known to be affected in AD dementia, subtle but reliable atrophy is identifiable in asymptomatic individuals nearly a decade before dementia, making this measure a potentially important imaging biomarker of early diagnosis (Dickerson et al., 2011). Volume losses in the medial temporal lobe (MTL) region—composed by the hippocampus and the parahippocampal gyrus—and posterior cingulated and orbitofrontal regions have been observed in AD and confirmed in many studies (Kesslak et al., 1991; Parnetti et al., 1996; Smith and Jobst, 1996; de Leon et al., 1997; Jack et al., 1997; Nagy et al., 1999; Bouwman et al., 2007; Eckerstrom et al., 2010; Jack et al., 2010; Zhang et al., 2010; Apostolova et al., 2012; Ewers et al., 2012; Leung et al., 2013; Heister et al.). This leads to a predictable pattern of brain atrophy that could be very useful to improve diagnosis and follow up and help making a better assessment of the neuroprotective effects of a therapy. The quantification of atrophy in the MTL (MTA) has been attempted using several different neuroimaging measurements, including rating scales, linear measurements, and volumetric methods.

Visual assessment rating scales are quick, and can be performed on large numbers of scans in a clinical setting, the disadvantage being that there is a loss of accuracy compared with objective analysis and are subjected to interrater variability (Westman et al., 2011). Some studies found that visual rating assessment of the MTL gave similar prediction accuracy to multivariate classification and manual hippocampal volumes (Ringman et al., 2010; Duara et al., 2013) while others reported the visual rating assessment failed to detect patients at high risk, such as people carrying mutations of familial AD and also failed to detect progression over time (Ridha et al., 2007; Pereira et al., 2013). In addition, clinical, demographic, and genetic variables can influence the classification of MTA cut-off scores, leading to misdiagnosis in some cases. These variables, in addition to the differential sensitivity and specificity of each cut-off, should be carefully considered when performing visual MTA assessment (Scheltens et al., 1992).

Linear measures of brain regions are easy to take using clinically available neuroimaging. Some studies attempted to define sentinel changes that will allow the use of linear measurements of the hippocampus or the temporal horn to support clinical decision making. These studies have yielded variable results, with sensitivities ranging from 33 to 93% and specificity of approximately 95% (Dahlbeck et al., 1991; Erkinjuntti et al., 1993; Frisoni et al., 2002).

Volumetric analysis provides an accurate and detailed measure of a predetermined circumscribed area or region of interest. For AD, the most used structure is the whole hippocampus. Some indices comparing the extent of atrophy in the hippocampus with the whole brain atrophy are also being described (http://brainatrophyindices.blogspot.com). Manual volumetry is considered the gold standard but it has some drawbacks. First it requires training since the tracer must learn to delineate the hippocampus's boundaries and anterior- and posterior-limits. Then segmentation of the hippocampus takes approximately 20–30 min, depending on user experience (Soininen et al., 1994; Petrella et al., 2003), which limits routine clinical use. Some groups automated segmentation techniques and protocols for multi-atlas driven automatic segmentation of the hippocampus (Morra et al., 2008; Brewer et al., 2009; Kovacevic et al., 2009). Results of a study comparing manual and automated determination of hippocampal volumes in MCI and early AD indicated that these two methods derived highly correlated results with strong agreement (Shen et al., 2010). Albeit homogenization efforts are under development (Frisoni and Jack, 2011; Boccardi et al., 2013), the complexity and diversity of protocols used for volumetry keeps being a limitation today.

In summary, despite convenience and strong correlation to severity of AD pathology, MTA is not being used in daily clinical practice for diagnosing prodromal and probable AD yet, as it is in clinical trials and research studies. This is mainly because the methods already described lack accuracy (visual methods) or are not convenient enough to be routinely used by clinicians in busy departments (volumetric methods).

## Purpose

In this report we aim to describe a new, objective and simple 2D-method for measuring atrophy of the MTL using clinically available neuroimaging. We also aim to explain the rationale behind this method. However, we do not seek to describe here the validity of this parameter for diagnosing AD since these researches are being conducted currently and results will be addressed in future publications.

## Protocol description

**Figure 1:**
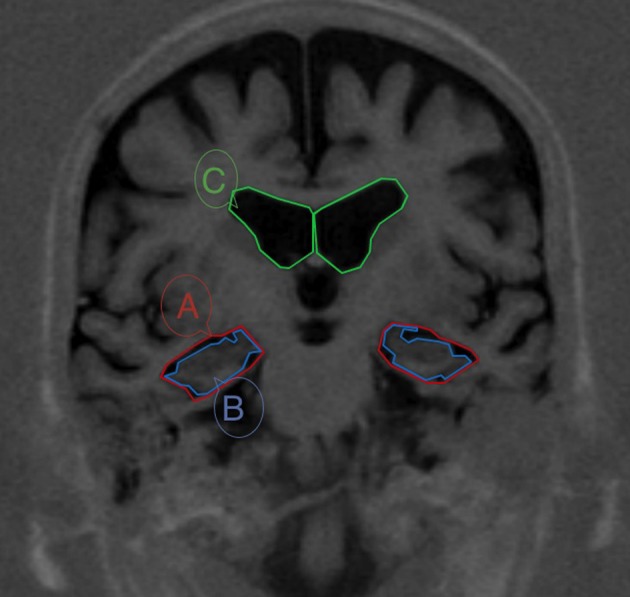
**MRI from a patient with MCI with boundaries of the three areas needed for calculating the Medial Temporal Atrophy index (MTAi)**. The section passes through the interpeduncular fosae. The three areas are: (1) the medial temporal lobe region **(A)**, defined in a coronal brain slide as the space bordered in its inferior side by the tentorium cerebelli, in its medial side by the cerebral peduncles, in its upper side by the roof of the temporal horn of the lateral ventricle and in its lateral side by the colateral sulcus and a straight-line linking the colateral sulcus with the lateral edge of the temporal horn of the lateral ventricle; (2) the parenchima within the medial temporal region, that includes the hippocampus and the parahippocampal girus **(B)**; and (3) the body of the ipsilateral lateral ventricle **(C)**.

**Figure 2:**
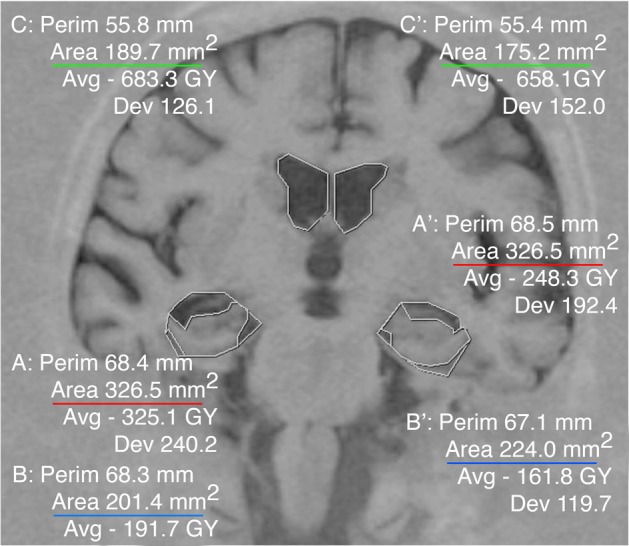
**Example of the Medial Temporal Atrophy index (MTAi) in a patient with mild AD**. The three areas were traced manually on each hemisphere using the software for visualizing radiological images IMPAX. The data needed to compute the index are displayed automatically. We have underlined the different areas in colors as in Figure 1. The MTAi in the right hemisphere is: rMTAi = (326.5 − 201.4) × 10/189.7 = 6.59. The MTAi in the left hemisphere is: lMTAi = (326.5 − 224.0) × 10/175.2 = 5.85. Note how in spite of the coincidence of this case with exactly the same medial temporal region **(A and A')** in both hemispheres, the right MTAi is clearly higher than the left MTAi. Indeed when we calculate the Index of Asymmetry (IA), it is higher than 3: IA = (5, 85 − 6.59)/(5, 85 + 6.59) × 100 = −5, 15%.

This method consists of measuring the area of 3 brain regions on one single MRI slide and then use these data for calculating a simple ratio. First, we take the coronal slide at the level of the interpeduncular fossa on the TIR sequence. Then, regions are traced manually, simply using the pointer-rule tool of any software for visualizing DICOM images. As guidelines to draw structures and boundaries we followed the atlases by Mai et al. (1997) and Duvernoy (1998). The three areas are: (1) the MTL region (A), defined in a coronal brain slide as the four-sided space bordered in its inferior side by the tentorium cerebelli, in its medial side by the cerebral peduncles, in its upper side by the roof of the temporal horn of the lateral ventricle and in its lateral side by the collateral sulcus and a straight-line linking the collateral sulcus with the lateral edge of the temporal horn of the lateral ventricle; (2) the parenchima within the medial temporal region, that includes the hippocampus and the parahippocampal gyrus—the fimbria taenia and plexus choroideus are excluded—(B); and (3) the body of the ipsilateral lateral ventricle (C) (Figure 1). Therefrom, we can compute the ratio “Medial Temporal Atrophy index (MTAi)” at both sides as follows: *MTAi* = (*A – B*) × 10/*C*. An example is shown in Figure 2.

If we have two MRI studies from different times (1 = first one, 2 = second one), we can also compute the yearly rate of MTA as follows: *yrMTA* = (*A2* − *B2*) − (*A1* − *B1*) × 120/(*#months between MRI studies*) and the yearly rate of relative MTA as follows: (*yrMTAr*) = (*A2* − *B2*) − (*A1* − *B1*) × 120/(*C2* − *C1*) × (*#months between MRI studies*).

## Expressing the medial temporal atrophy index

When we compute the MTAi we obtain 2 values, one for each hemisphere. In addition, it is also interesting to compute the median of these 2 values, and the index of asymmetry (IA). We determine the IA using formula *IA* = (*lMTAi* − *dMTAi*)/(*lMTAi* + *dMTAi*) × 100. Small positive or negative IA values of magnitude less than ~±3% indicate that there is not a significant hemispheric asymmetry and the median MTAi can be used alone as a parameter of the global relative MTA. Higher IA values indicate significant hemispheric asymmetry and the median value should not be used alone since it is not a good representative value of the extent of relative MTA. Thus, the MTAi can be presented directly as the absolute right/left MTAi values or as the median MTAi with the IA (Table 1).

**Table 1:** **Mean of the mean Medial Temporal Atrophy index (mMTAi) and Index of Asymmetry (IA) values in short series of patients with MCI (3), mild AD (3), moderate AD (3), severe AD (3), FTLD -not staged- (3), LBD -not staged- (3) and 5 healthy controls. Values are merely illustrative -not informative-**.

	**Mean mMTAi**	**Mean AI**
Healthy control	2, 4	1, 8
MCI	3, 1	2, 4
Mild AD	4, 6	2, 6
Moderate AD	5, 2	2, 8
Severe AD	5, 8	3, 4
FTLD	3, 8	8, 2
DLB	2, 7	3, 7

## Rationale behind the medial temporal atrophy index

The rationale behind this method is based on two premises: First, AD is a disease affecting the hippocampus, not a disease of the hippocampus. From a neuropathological point of view it is evident that that the characteristic pathological changes in AD begin outside the hippocampus, with development of neurofibrillary tangles in the transentorhinal and entorhinal cortex, spreading subsequently to the subiculum and CA1 regions of the hippocampus (Jack et al., 1992; Braak and Braak, 1985; Convit et al., 2000; Kerchner et al., 2010; Lim et al., 2012) and later to limbic, and ultimately to neocortical regions, such as the precuneus, middle frontal gyrus, and posterior cingulate gyrus. The severity of this atrophy, at least in the medial temporal regions, correlates with the severity of underlying neuropathological changes seen on postmortem studies (Echávarri et al., 2011). The second premise is that, despite most volumetric methods focus on the hippocampus and disregard the parahippocampal gyrus, many studies have shown that parahippocampal atrophy is as good indicator of AD as the hippocampus atrophy is (Nestor et al., 2008; Burgmans et al., 2011; Smith et al., 2012; Zarei et al., 2012). Thus, the entorinal cortex, the hippocampus, and the parahippocampal gyrus may be considered as the “epicentrum” of the neurodegenerative process. Therefore, in order to pick up the disease early we do not need to find out the volume of the whole hippocampus but detect atrophy at “the point” where the pathology is visible first.

### The slice selected

Functionally, the hippocampus can be segmented into three distinct anatomical and functional subregions (head, body, and tail), according to the morphology and relative connectivity with prefrontal cortex (PFC), posterior cingulate cortex (PCC), and thalamus, respectively. The AD group show stronger hippocampus–PFC and weaker hippocampus–PCC functional connectivity, the magnitudes of which correlate with cognitive performance (Convit et al., 2000; Dickerson et al., 2011; Libby et al., 2012). In line with this fact and in order to assess the body of the hippocampus we have taken the coronal section passing through the interpeduncular fossa where the body of the hippocampus can be clearly viewed. However, this index might be performed on any other coronal slide where the MTL structures are viewed.

### The areas selected

Age-associated differences are detected in the MTL (Parnetti et al., 1996; Jack et al., 1997; Apostolova et al., 2012; Leung et al., 2013) with an acceleration of MTA starting around 72 years of age in healthy people (Jack et al., 1997). However, these changes are modest and their rate of progression over time is relatively slow with a mean rate of about 1.6% per year (Leung et al., 2013). Accelerated MTA is a consistent finding in AD and MCI with rates of about 2.8% in stable MCI, 3.7% in MCI transitioning to AD (MCI progressors), and up to 4.0% in AD (Kesslak et al., 1991; Parnetti et al., 1996; Jack et al., 1997; Bouwman et al., 2007; Eckerstrom et al., 2010; Jack et al., 2010; Apostolova et al., 2012; Ewers et al., 2012; Leung et al., 2013; Heister et al.). Frontotemporal dementia may also lead to MTA, but in a different pattern: frontotemporal dementia and semantic dementia show atrophy in the anterior portion of the hippocampus, and in semantic dementia the atrophy is asymmetrical, with the left hippocampus being affected more severely. No significant hippocampal atrophy is detected in non-fluent progressive aphasia (Barber et al., 1999; Schacter and Wagner, 1999; Chan et al., 2001; van de Pol et al., 2006). Other diseases such as dementia with Lewy bodies do not show MTA or it is much milder (Hashimoto et al., 1998; Whitwell et al., 2007; Chou et al., 2010).

In contrast to MTA, ventricular enlargement (body of lateral ventricles) in old people lacks specificity representing a measure of global brain atrophy due to aging or any neurodegenerative disorder. Global ventricular enlargement correlates with decline in cognitive performance and with cerebrospinal fluid pathologic markers of AD (Thompson et al., 2004; Apostolova et al., 2010). Absolute ventricular volumes and ventricular enlargement are greater in subjects with AD and MCI compared to age-matched controls. Ventricular enlargement also demonstrated sensitivity to disease progression by way of discriminating between subjects with stable MCI and those that progressed to AD (Nestor et al., 2008). However, it is important to note that all these studies were made using absolute ventricular volumes, without differentiation among the different portions of the lateral ventricles, while the lateral (temporal) horns are the portion contributing most to the ventricular enlargement in early AD (Giesel et al., 2006). It is well-known that enlargement of lateral ventricles is a measure of unspecific global brain atrophy since it is strongly associated both with aging in healthy and with neurodegeneration (Apostolova et al., 2012). Almost any neurodegenerative disorder affecting the brain hemispheres leads to some degree of ventricular enlargement, including Parkinson's disease (Meyer et al., 2007; Apostolova et al., 2010; Dalaker et al., 2011), Lewy-Bodies Dementia (Meyer et al., 2007), Frontotemporal Lobe Dementia (Galton et al., 2001; Gordon et al., 2010), and Corticobasal Degeneration (Hauser et al., 1996) and so do some psychiatric conditions (Swayze et al., 1990; Mathalon et al., 2001). Thus, it would be interesting to compare the extent of atrophy in the MTL with the extent of global brain atrophy (Table 1).

### The ratio

This index reflects the rate of atrophy in the MTL—that is a value rather specific of AD since its early stages—in relation to the global unspecific atrophy represented by ventricular enlargement. Thus, it is a measure estimative of the contribution of the atrophy in the MTL to the whole brain atrophy.

## Advantages and limitations of the MTA index

From the clinician's point of view, the MTA index has the following advantages over other methods: (1) Measurement and scoring of MTA index is objective and reliable, providing a distinct advantage over visual techniques. (2) Volumetric measurements require the use of special software, and much greater technical stringency in the acquisition of the MRI scans and are far more prone to a variety of measurement errors. Delineating the areas needed for calculating the MTA index is fast and easy; little training is needed. Therefore, it can be implemented for daily clinical practice using basic neuroimaging facilities currently available in most hospitals with busy clinical settings. (3) An additional advantage of using MTA index over volumetric measures is that regional brain volumes are variable across individuals and need to be normalized by conversion to a ratio of the absolute volumes to intracranial volume, whereas the MTA index has built-in normalization and thus avoids multiplicative errors inherent in using ratios of two quantitative variables. (4) The same way, as aging affects both the hippocampus and lateral ventricles independent of AD pathology, aging should be included as covariate in methods providing absolute volumes or scores. The MTA index is an “intra-patient” ratio comparing the MTL and lateral ventricles, so it will probably not need cut-off scores adjusted by age. For the yearly rate of MTA and the yearly rate of relative MTA, normalization is not necessary neither because each subject serves as their own control.

On the other hand, the main limitation of the MTA index is that scoring is based on measurements performed on a single coronal slice, thereby providing a limited perspective of overall brain pathology. It is also expected that other conditions affecting the ventricular morphology, such as hydrocephalus, will probably alter the interpretation of the MTAi in these cases.

This paper is a methodological description only. Cut-off scores have to be calculated and its use as a parameter for diagnosing AD in research and clinical practice has to be validated. Particularly, prospective studies are needed to assess the usefulness of MTA index in the diagnosis of early AD, in tracking the progression of AD and in the differential diagnosis of AD with other dementias.

## Conclusions

We report a new, manual method for assessing medial temporal lobe atrophy (MTA) that is objective and easy to apply using clinically available neuroimaging. It may have some advantages over visual and volumetric methods that still need to be evaluated.

### Conflict of interest statement

The authors declare that the research was conducted in the absence of any commercial or financial relationships that could be construed as a potential conflict of interest.

## References

[B1] Albert M. S. Dekosky S. T. Dickson D. Dubois B. Feldman H. H. Fox N. C. (2011). The diagnosis of mild cognitive impairment due to Alzheimer's disease: recommendations from the National Institute on Aging and Alzheimer's Association workgroup. Alzheimers Dement. 7, 270–279 10.1016/j.jalz.2011.03.008 21514249 PMC3312027

[B2] Apostolova L. G. Beyer M. Green A. E. Hwang K. S. Morra J. H. Chou Y. Y. (2010). Hippocampal, caudate, and ventricular changes in Parkinson's disease with and without dementia. Mov. Disord. 25, 687–695 10.1002/mds.22799 20437538 PMC3068920

[B3] Apostolova L. G. Green A. E. Babakchanian S. Hwang K. S. Chou Y. Y. Toga A. W. (2012). Hippocampal atrophy and ventricular enlargement in normal aging, mild cognitive impairment (MCI), and Alzheimer disease. Alzheimer Dis. Assoc. Disord. 26, 17–27 10.1097/WAD.0b013e3182163b62 22343374 PMC3286134

[B4] Barber R. Gholkar A. Scheltens P. Ballard C. McKeith I. G. O'Brien J. T. (1999). Medial temporal lobe atrophy on MRI in dementia with Lewy bodies. Neurology 52, 1153–1158 10.1212/WNL.52.6.1153 10214736

[B5] Boccardi M. Bocchetta M. Apostolova L. G. Preboske G. Robitaille N. Pasqualetti P. (2013). Establishing magnetic resonance images orientation for the EADC-ADNI manual hippocampal segmentation protocol. J. Neuroimaging. [Epub ahead of print]. 10.1111/jon.12065 24279479

[B6] Bouwman F. H. Schoonenboom S. N. van der Flier W. M. van Elk E. J. Kok A. Barkhof F. (2007). CSF biomarkers and medial temporal lobe atrophy predict dementia in mild cognitive impairment. Neurobiol. Aging 28, 1070–1074 10.1016/j.neurobiolaging.2006.05.006 16782233

[B7] Braak H. Braak E. (1985). On areas of transition between entorhinal allocortex and temporal isocortex in the human brain. Normal morphology and lamina-specific pathology in Alzheimer's disease. Acta Neuropathol. 68, 325–332 10.1007/BF00690836 4090943

[B8] Brewer J. B. Magda S. Airriess C. Smith M. E. (2009). Fully automated quantification of regional brain volumes for improved detection of focal atrophy in Alzheimer disease. AJNR Am. J. Neuroradiol. 30, 578–580 10.3174/ajnr.A1402 19112065 PMC5947999

[B9] Burgmans S. van Boxtel M. P. van den Berg K. E. Gronenschild E. H. Jacobs H. I. Jolles J. (2011). The posterior parahippocampal gyrus is preferentially affected in age-related memory decline. Neurobiol. Aging 32, 1572–1578 10.1016/j.neurobiolaging.2009.09.008 19879667

[B10] Chan D. Fox N. C. Scahill R. I. Crum W. R. Whitwell J. L. Leschziner G. (2001). Patterns of temporal lobe atrophy in semantic dementia and Alzheimer's disease. Ann. Neurol. 49, 433–442 10.1002/ana.92 11310620

[B11] Chou Y. Y. Leporé N. Saharan P. Madsen S. K. Hua X. Jack C. R. (2010). Alzheimer's disease neuroimaging initiative. Ventricular maps in 804 ADNI subjects: correlations with CSF biomarkers and clinical decline. Neurobiol. Aging 31, 1386–1400 10.1016/j.neurobiolaging.2010.05.001 20620663 PMC2904619

[B12] Convit A. de Asis J. de Leon M. J. Tarshish C. Y. De Santi S. Rusinek H. (2000). Atrophy of the medial occipitotemporal, inferior, and middle temporal gyri in non-demented elderly predict decline to Alzheimer's disease. Neurobiol. Aging 21, 19–26 10.1016/S0197-4580(99)00107-4 10794844

[B13] Dahlbeck J. W. McCluney K. W. Yeakley J. W. Fenstermacher M. J. Bonmati C. Van Horn G. (1991). The interuncal distance: a new MR measurement for the hippocampal atrophy of Alzheimer disease. AJNR Am. J. Neuroradiol. 12, 931–932 1950924 PMC8333519

[B14] Dalaker T. O. Zivadinov R. Ramasamy D. P. Beyer M. K. Alves G. Bronnick K. S. (2011). Ventricular enlargement and mild cognitive impairment in early Parkinson's disease. Mov. Disord. 26, 297–301 10.1002/mds.23443 21412836

[B15] de Leon M. J. George A. E. Golomb J. Tarshish C. Convit A. Kluger A. (1997). Frequency of hippocampal formation atrophy in normal aging and Alzheimer's disease. Neurobiol. Aging 18, 1–11 10.1016/S0197-4580(96)00213-8 8983027

[B16] Dickerson B. C. Stoub T. R. Shah R. C. Sperling R. A. Killiany R. J. Albert M. S. (2011). Alzheimer-signature MRI biomarker predicts AD dementia in cognitively normal adults. Neurology 76, 1395–1402 10.1212/WNL.0b013e3182166e96 21490323 PMC3087406

[B17] Duara R. Loewenstein D. A. Shen Q. Barker W. Varon D. Greig M. T. (2013). Volumetric and visual ratings of medical temporal atrophy in AD and MCI: comparison of age-specific cut-offs. Front. Aging Neurosci. 5:47 10.3389/fnagi.2013.00047 24065917 PMC3776563

[B18] Duvernoy H. M. (1998). The Human Hippocampus: Functional Anatomy, Vascularization and Serial Sections with MRI. Berlin: Springer-Verlag. 10.1007/978-3-662-03628-0

[B19] Echávarri C. Aalten P. Uylings H. B. Jacobs H. I. Visser P. J. Gronenschild E. H. (2011). Atrophy in the parahippocampal gyrus as an early biomarker of Alzheimer's disease. Brain Struct. Funct. 215, 265–271 10.1007/s00429-010-0283-8 20957494 PMC3041901

[B20] Eckerstrom C. Andreasson U. Olsson E. Rolstad S. Blennow K. Zetterberg H. (2010). Combination of hippocampal volume and cerebrospinal fluid biomarkers improves predictive value in mild cognitive impairment. Dement. Geriatr. Cogn. Disord. 29, 294–300 10.1159/000289814 20389071

[B21] Erkinjuntti T. Lee D. H. Gao F. Steenhuis R. Eliasziw M. Fry R. (1993). Temporal lobe atrophy on magnetic resonance imaging in the diagnosis of early Alzheimer's disease. Arch. Neurol. 50, 305–310 10.1001/archneur.1993.00540030069017 8442711

[B22] Ewers M. Walsh C. Trojanowski J. Q. Shaw L. M. Petersen R. C. Jack C. R. (2012). Prediction of conversion from mild cognitive impairment to Alzheimer's disease dementia based upon biomarkers and neuropsychological test performance. Neurobiol. Aging 33, 1203–1214 10.1016/j.neurobiolaging.2010.10.019 21159408 PMC3328615

[B24] Frisoni G. B. Geroldi C. Beltramello A. Bianchetti A. Binetti G. Bordiga G. (2002). Radial width of the temporal horn: a sensitive measure in Alzheimer disease. AJNR Am. J. Neuroradiol. 23, 35–47 11827874 PMC7975508

[B23] Frisoni G. B. Jack C. R. (2011). Harmonization of magnetic resonance-based manual hippocampal segmentation: a mandatory step for wide clinical use. Alzheimers Dement. 7, 171–174 10.1016/j.jalz.2010.06.007 21414554

[B25] Galton C. J. Gomez-Anson B. Antoun N. Scheltens P. Patterson K. Graves M. (2001). Temporal lobe rating scale: application to Alzheimer's disease and frontotemporal dementia. J. Neurol. Neurosurg. Psychiatr. 70, 165–173 10.1136/jnnp.70.2.165 11160463 PMC1737195

[B26] Giesel F. L. Hahn H. K. Thomann P. A. Widjaja E. Wignall E. von Tengg-Kobligk H. (2006). Temporal horn index and volume of medial temporal lobe atrophy using a new semiautomated method for rapid and precise assessment. AJNR Am. J. Neuroradiol. 27, 1454–1458 16908557 PMC7977513

[B27] Gordon E. Rohrer J. D. Kim L. G. Omar R. Rossor M. N. Fox N. C. (2010). Measuring disease progression in frontotemporal lobar degeneration: a clinical and MRI study. Neurology 74, 666–673 10.1212/WNL.0b013e3181d1a879 20177120 PMC2830919

[B28] Hashimoto M. Kitagaki H. Imamura T. Hirono N. Shimomura T. Kazui H. (1998). Medial temporal and whole-brain atrophy in dementia with Lewy bodies: a volumetric MRI study. Neurology 51, 357–362 10.1212/WNL.51.2.357 9710003

[B29] Hauser R. A. Murtaugh F. R. Akhter K. Gold M. Olanow C. W. (1996). Magnetic resonance imaging of corticobasal degeneration. J. Neuroimaging 6, 222–226 8903073 10.1111/jon199664222

[B30] Heister D. Brewer J. B. Magda S. Blennow K. McEvoy L. K. Alzheimer's Disease Neuroimaging Initiative. (2011). Predicting MCI outcome with clinically available MRI and CSF biomarkers. Neurology 77, 1619–1628 10.1212/WNL.0b013e3182343314 21998317 PMC3198979

[B31] Jack C. R. Jr. Petersen R. C. O'Brien P. C. Tangalos E. G. (1992). MR-based hippocampal volumetry in the diagnosis of Alzheimer's disease. Neurology 42, 183–188 1734300 10.1212/wnl.42.1.183

[B32] Jack C. R. Jr. Petersen R. C. Xu Y. C. Waring S. C. O'Brien P. C. Tangalos E. G. (1997). Medial temporal atrophy on MRI in normal aging and very mild Alzheimer's disease. Neurology 49, 786–794 10.1212/WNL.49.3.786 9305341 PMC2730601

[B33] Jack C. R. Jr. Wiste H. J. Vemuri P. Weigand S. D. Senjem M. L. Zeng G. (2010). Brain beta-amyloid measures and magnetic resonance imaging atrophy both predict time-to-progression from mild cognitive impairment to Alzheimer's disease. Brain 133, 3336–3348 10.1093/brain/awq277 20935035 PMC2965425

[B34] Kerchner G. A. Hess C. P. Hammond-Rosenbluth K. E. Xu D. Rabinovici G. D. Kelley D. A. (2010). Hippocampal CA1 apical neuropil atrophy in mild Alzheimer disease visualized with 7-T MRI. Neurology 75, 1381–1387 10.1212/WNL.0b013e3181f736a1 20938031 PMC3013485

[B35] Kesslak J. P. Nalcioglu O. Cotman C. W. (1991). Quantification of magnetic resonance scans for hippocampal and parahippocampal atrophy in Alzheimer's disease. Neurology 41, 51–54 10.1212/WNL.41.1.51 1985296

[B36] Kovacevic S. Rafii M. S. Brewer J. B. (2009). High-throughput, fully automated volumetry for prediction of MMSE and CDR decline in mild cognitive impairment. Alzheimer Dis. Assoc. Disord. 23, 139–145 10.1097/WAD.0b013e318192e745 19474571 PMC2688740

[B37] Leung K. K. Bartlett J. W. Barnes J. Manning E. N. Ourselin S. Fox N. C. (2013). Azheimer's disease neuroimaging initiative. Cerebral atrophy in mild cognitive impairment and Alzheimer disease: rates and acceleration. Neurology 80, 648–654 10.1212/WNL.0b013e318281ccd3 23303849 PMC3590059

[B38] Libby L. Ekstrom A. Ragland J. D. Ranganath C. (2012). Differential connectivity of perirhinal and parahippocampal cortices within human hippocampal subregions revealed by high-resolution functional imaging. J. Neurosci. 32, 6550–6560 10.1523/JNEUROSCI.3711-11.2012 22573677 PMC3374643

[B39] Lim H. K. Jung W. S. Ahn K. J. Won W. Y. Hahn C. Lee S. Y. (2012). Relationships between hippocampal shape and cognitive performances in drug-naïve patients with Alzheimer's disease. Neurosci. Lett. 516, 124–129 10.1016/j.neulet.2012.03.072 22490885

[B40] Mai J. K. Assheuer J. Paxinos G. (1997). Atlas of the Human Brain. San Diego, CA: Academic Press

[B41] Mathalon D. H. Sullivan E. V. Lim K. O. Pfefferbaum A. (2001). Progressive brain volume changes and the clinical course of Schizophrenia in men: a longitudinal magnetic resonance imaging study. Arch. Gen. Psychiatry 58, 148–157 10.1001/archpsyc.58.2.148 11177116

[B42] McKhann G. M. Knopman D. S. Chertkow H. Hyman B. T. Jack C. R. Jr. Kawas C. H. (2011). The diagnosis of dementia due to Alzheimer's disease: recommendations from the National Institute on Aging-Alzheimer's Association workgroups on diagnostic guidelines for Alzheimer's disease. Alzheimers Dement. 7, 263–269 10.1016/j.jalz.2011.03.005 21514250 PMC3312024

[B43] Meyer J. S. Huang J. Chowdhury M. H. (2007). MRI confirms mild cognitive impairments prodromal for Alzheimer's, vascular and Parkinson-Lewy body dementias. J. Neurol. Sci. 257, 97–104 10.1016/j.jns.2007.01.016 17316690

[B44] Morra J. H. Tu Z. Apostolova L. G. Green A. E. Avedissian C. Madsen S. K. (2008). Alzheimer's disease neuroimaging initiative. Validation of a fully automated 3D hippocampal segmentation method using subjects with Alzheimer's disease mild cognitive impairment, and elderly controls. Neuroimage 43, 59–68 10.1016/j.neuroimage.2008.07.003 18675918 PMC2624575

[B45] Morris J. C. Storandt M. McKeel D. W. Jr. Rubin E. H. Price J. L. Grant E. A. (1996). Cerebral amyloid deposition and diffuse plaques in “normal” aging: evidence for presymptomatic and very mild Alzheimer's disease. Neurology 46, 707–719 10.1212/WNL.46.3.707 8618671

[B46] Nagy Z. Hindley N. J. Braak H. Braak E. Yilmazer-Hanke D. M. Schultz C. (1999). The progression of Alzheimer's disease from limbic regions to the neocortex: clinical, radiological and pathological relationships. Dement. Geriatr. Cogn. Disord. 10, 115–120 10.1159/000017111 10026385

[B47] Nestor S. M. Rupsingh R. Borrie M. Smith M. Accomazzi V. Wells J. L. (2008). Alzheimer's disease neuroimaging initiative. Ventricular enlargement as a possible measure of Alzheimer's disease progression validated using the Alzheimer's disease neuroimaging initiative database. Brain 131, 2443–2454 10.1093/brain/awn146 18669512 PMC2724905

[B48] Parnetti L. Lowenthal D. T. Presciutti O. Pelliccioli G. P. Palumbo R. Gobbi G. (1996). 1H-MRS, MRI-based hippocampal volumetry, and 99mTc-HMPAO-SPECT in normal aging, age-associated memory impairment, and probable Alzheimer's disease. J. Am. Geriatr. Soc. 44, 133–138 8576501 10.1111/j.1532-5415.1996.tb02428.x

[B49] Pereira J. B. Cavallin L. Spulber G. Aguilar C. Mecocci P. Vellas B. (2013). Influence of age, disease onset and ApoE4 on visual medial temporal lobe atrophy cut-offs. J. Intern. Med. [Epub ahead of print]. 10.1111/joim.12148 24118559

[B50] Petrella J. R. Coleman R. E. Doraiswamy P. M. (2003). Neuroimaging and early diagnosis of Alzheimer disease: a look to the future. Radiology 226, 315–336 10.1148/radiol.2262011600 12563122

[B51] Ridha B. H. Barnes J. van de Pol L. A. Schott J. M. Boyes R. G. Siddique M. M. (2007). Application of automated medial temporal lobe atrophy scale to Alzheimer disease. Arch. Neurol. 64, 849–854 10.1001/archneur.64.6.849 17562933

[B52] Ringman J. M. Pope W. Salamon N. (2010). Insensitivity of visual assessment of hippocampal atrophy in familial Alzheimer's disease. J. Neurol. 257, 839–842 10.1007/s00415-009-5436-4 20047059 PMC2864895

[B53] Schacter D. L. Wagner A. D. (1999). Medial temporal lobe activations in fMRI and PET studies of episodic encoding and retrieval. Hippocampus 9, 7–24 10.1002/(SICI)1098-1063(1999)9:1%3C7::AID-HIPO2%3E3.0.CO;2-K 10088896

[B54] Scheltens P. Leys D. Barkhof F. Huglo D. Weinstein H. C. Vermersch P. (1992). Atrophy of medial temporal lobes on MRI in “probable” Alzheimer's disease and normal ageing: diagnostic value and neuropsychological correlates. J. Neurol. Neurosurg. Psychiatr. 55, 967–972 10.1136/jnnp.55.10.967 1431963 PMC1015202

[B55] Shen L. Saykin A. J. Kim S. Firpi H. A. West J. D. Risacher S. L. (2010). Comparison of manual and automated determination of hippocampal volumes in MCI and early AD. Brain Imaging Behav. 4, 86–95 10.1007/s11682-010-9088-x 20454594 PMC2863347

[B56] Smith A. D. Jobst K. A. (1996). Use of structural imaging to study the progression of Alzheimer's disease. Br. Med. Bull. 52, 575–586 10.1093/oxfordjournals.bmb.a011568 8949258

[B57] Smith C. D. Andersen A. H. Gold B. T. (2012). Structural brain alterations before mild cognitive impairment in ADNI: validation of volume loss in a predefined antero-temporal region. J. Alzheimers. Dis. 31, S49–S58 10.3233/JAD-2012-120157 22460332 PMC3652624

[B58] Soininen H. S. Partanen K. Pitkanen A. Vainio P. Hanninen T. Hallikainen M. (1994). Volumetric MRI analysis of the amygdala and the hippocampus in subjects with age-associated memory impairment: correlation to visual and verbal memory. Neurology 44, 1660–1668 10.1212/WNL.44.9.1660 7936293

[B59] Sperling R. A. Aisen P. S. Beckett L. A. Bennett D. A. Craft S. Fagan A. M. (2011). Toward defining the preclinical stages of Alzheimer's disease: recommendations from the National Institute on Aging-Alzheimer's Association workgroups on diagnostic guidelines for Alzheimer's disease. Alzheimers Dement. 7, 280–292 10.1016/j.jalz.2011.03.003 21514248 PMC3220946

[B60] Swayze V. W. II. Andreasen N. C. Alliger R. J. Ehrhardt J. C. Yuh W. C. (1990). Structural brain abnormalities in bipolar affective disorder: ventricular enlargement and focal signal hyperintensities. Arch. Gen. Psychiatry 47, 1054–1059 10.1001/archpsyc.1990.01810230070011 2241506

[B61] Thompson P. M. Hayashi K. M. De Zubicaray G. I. Janke A. L. Rose S. E. Semple J. (2004). Mapping hippocampal and ventricular change in Alzheimer disease. Neuroimage 22, 1754–1766 10.1016/j.neuroimage.2004.03.040 15275931

[B62] van de Pol L. A. Hensel A. van der Flier W. M. Visser P. J. Pijnenburg Y. A. Barkhof F. (2006). Hippocampal atrophy on MRI in frontotemporal lobar degeneration and Alzheimer's disease. J. Neurol. Neurosurg. Psychiatr. 77, 439–442 10.1136/jnnp.2005.075341 16306153 PMC2077497

[B63] Westman E. Cavallin L. Muehlboeck J. S. Zhang Y. Mecocci P. Vellas B. (2011). AddNeuroMed consortium. Sensitivity and specificity of medial temporal lobe visual ratings and multivariate regional MRI classification in Alzheimer's disease. PLoS ONE 6:e22506 10.1371/journal.pone.0022506 21811624 PMC3141068

[B64] Whitwell J. L. Weigand S. D. Shiung M. M. Boeve B. F. Ferman T. J. Smith G. E. (2007). Focal atrophy in dementia with Lewy bodies on MRI: a distinct pattern from Alzheimer's disease. Brain 130(Pt 3), 708–719 10.1093/brain/awl388 17267521 PMC2730778

[B65] Zarei M. Beckmann C. F. Binnewijzend M. A. Schoonheim M. M. Oghabian M. A. Sanz-Arigita E. J. (2012). Functional segmentation of the hippocampus in the healthy human brain and in Alzheimer's disease. Neuroimage 66C, 28–35 10.1016/j.neuroimage.2012.10.071 23128076

[B66] Zhang Y. Qiu C. Lindberg O. Bronge L. Aspelin P. Bäckman L. (2010). Acceleration of hippocampal atrophy in a non-demented elderly population: the SNAC-K study. Int. Psychogeriatr. 22, 14–25 10.1017/S1041610209991396 19958567

